# Immunogenicity and safety of high-dose quadrivalent influenza vaccine in Japanese adults ≥65 years of age: a randomized controlled clinical trial

**DOI:** 10.1080/21645515.2019.1677437

**Published:** 2019-11-19

**Authors:** Leilani Sanchez, Osamu Matsuoka, Satoshi Inoue, Takahiro Inoue, Ya Meng, Takahiro Nakama, Kumiko Kato, Aseem Pandey, Lee-Jah Chang

**Affiliations:** a Research and Development, Sanofi Pasteur, Taguig, Philippines; b Medical Corporation Heishinkai ToCROM Clinic, Tokyo, Japan; c Medical Corporation Heishinkai OCROM Clinic, Osaka, Japan; d Sanofi K.K, Tokyo, Japan; e Sanofi Pasteur, Swiftwater, PA, USA

**Keywords:** Quadrivalent influenza vaccine, high-dose influenza vaccine, elderly adults, immunogenicity, safety, Japan, intramuscular, subcutaneous

## Abstract

A trivalent high-dose inactivated influenza vaccine has been licensed in healthy adults ≥65 years of age and provides better protection against influenza infection and related complications than trivalent standard-dose vaccine. This phase I/II clinical trial (NCT03233217), conducted at two sites in Japan, examined the safety and immunogenicity of a quadrivalent formulation of the high-dose inactivated influenza vaccine (IIV4-HD). Healthy adults ≥65 years of age were randomized to receive IIV4-HD by intramuscular injection (n = 60), IIV4-HD by subcutaneous injection (n = 60), or a quadrivalent standard-dose inactivated influenza vaccine (IIV4-SD) by subcutaneous injection (n = 55). Irrespective of administration route, post-vaccination (day 28–35) hemagglutination inhibition geometric mean titers and seroconversion rates were higher for IIV4-HD than for IIV4-SD. Hemagglutination inhibition geometric mean titers and seroconversion rates were also higher for intramuscular than subcutaneous administration of IIV4-HD. Solicited reactions were more common in participants who received IIV4-HD administered subcutaneously than in those who received IIV4-HD administered intramuscularly or IIV4-SD administered subcutaneously. Unsolicited adverse events were similar between the vaccine groups, and no safety signals were detected. This study showed that IIV4-HD administered by either intramuscular or subcutaneous injection was well tolerated and highly immunogenic in healthy Japanese adults ≥65 years of age. Although this study was descriptive, the results add to the evidence that high-dose inactivated influenza vaccines are more immunogenic than standard-dose vaccines in this age group and that intramuscular administration provides greater immunogenicity and lower reactogenicity than subcutaneous administration.

## Introduction

Most influenza-related hospitalizations and deaths occur in adults ≥65 years of age.^1^ This appears to be due to increasing comorbidities and waning immune responses associated with aging.^2,3^ Because of the increased risk, the World Health Organization and most national health authorities recommend that, along with young children, pregnant women, and individuals with certain underlying conditions, adults ≥65 years of age should be prioritized for influenza vaccination.^4^

Influenza vaccination is becoming increasingly important in Japan because it has one of the oldest and most rapidly aging populations globally.^5^ Routine vaccination against influenza was instituted in Japan in 2001 for adults ≥65 years of age and adults 60–64 years of age with respiratory, cardiac, or renal disease or infection with human immunodeficiency virus.^6,7^

Since the 2015/16 season, quadrivalent influenza vaccines have been used to vaccinate eligible individuals in Japan.^8^ Quadrivalent influenza vaccines contain antigen from two influenza A strains (A/H1N1 and A/H3N2) and both influenza B-strain lineages (Victoria and Yamagata), whereas trivalent vaccines contain the two A strains and a single B-lineage strain. Quadrivalent influenza vaccines were developed to avoid mismatches between the B-strain lineage in trivalent vaccines and the predominant circulating B lineage,^9^ which occurred in about one-quarter of influenza seasons between 2000 and 2013.^10^ Switching from trivalent to quadrivalent influenza vaccines in Japan has prevented an estimated 2030 hospitalizations and 98 deaths each year and saved an estimated 10.75 million US dollars from a societal perspective.^11^

To provide improved protection against influenza infection, a trivalent high-dose, split-virion inactivated influenza vaccine (IIV3-HD; Fluzone® High-Dose, Sanofi Pasteur)^12^ has been licensed in adults ≥65 years of age in the US since 2009, Canada since 2015, Australia since 2017, Brazil since 2018, and the United Kingdom since 2019. This vaccine contains 60 µg hemagglutinin per influenza strain, which is four times the antigen content of standard-dose influenza vaccines. A multicenter phase III trial in the US and Canada showed that, in adults ≥65 years of age, IIV3-HD was 24.2% more effective than a standard-dose trivalent influenza vaccine (IIV3-SD) in preventing laboratory-confirmed influenza caused by any strain and 35.4% more effective than IIV3-SD at preventing laboratory-confirmed influenza caused by vaccine-similar strains.^13^ IIV3-HD is well tolerated in adults ≥65 years of age, although as expected with the increased antigen dose, local reactions are more common with IIV3-HD.^14^ These findings have been supported by post-marketing studies, which have shown that IIV3-HD provides improved protection against influenza, serious pneumonia, post-influenza death, and all-cause, influenza-related, and cardiorespiratory-associated hospitalization.^15–19^

To further improve protection against influenza in older adults, a quadrivalent formulation of the high-dose inactivated influenza vaccine (IIV4-HD) is being developed. A recent phase III trial in the US showed that, in healthy adults ≥65 years of age, IIV4-HD was well tolerated and induced immune responses that were non-inferior to responses induced by IIV3-HD for the shared strains and superior responses for the additional B-lineage strain.^20^

The current study, performed in Japan during the 2017–2018 influenza season, compared the safety and immunogenicity of IIV4-HD and standard-dose quadrivalent inactivated influenza vaccine (IIV4-SD) in adults ≥65 years of age. In Japan, the standard of care for influenza vaccination is administration by subcutaneous (SC) injection, whereas the safety and efficacy of high-dose vaccines have so far been demonstrated following intramuscular (IM) injection. In this study, IIV4-HD was therefore administered by both IM and SC injection, whereas IIV4-SD was administered by SC injection.

Strains included in influenza vaccines are selected from the list of World Health Organization-recommended strains for each season, although the specific strains are selected by the Vaccines and Related Biological Products Advisory Committee for US vaccines and the National Institute of Infectious Diseases for Japanese vaccines. During the 2017–2018 influenza, three of four strains differed slightly between the US IIV4-HD and Japanese licensed IIV4-SD. This study therefore measured immunogenicity against all seven strains included in the two vaccines.

## Patients and methods

### Study design

This was a phase I/II, randomized, parallel, modified double-blind, multi-center study in adults ≥65 years of age (NCT03233217) conducted between September and November 2017 at two clinics in Japan (ToCROM Clinic, Tokyo, Japan and OCROM Clinic, Osaka, Japan). The study was approved by the independent ethics committee or institutional review board for each institution and conducted in compliance with the standards established by the Declaration of Helsinki, the International Conference for Harmonization guidelines for Good Clinical Practice, and all local and national regulations and directives. All participants provided signed, informed consent before taking part in the study. An abbreviated version of the study protocol is available at https://clinicaltrials.gov/ct2/show/NCT03233217. No amendments were made to the protocol.

### Participants

The study included adults ≥65 years of age not vaccinated against influenza in the preceding 6 months. Potential participants were excluded if they had any condition or were receiving any treatment that, in the opinion of the investigator, could interfere with the trial conduct, completion, or assessments. Other exclusions are listed in Supplementary table S1.

### Vaccines

IIV4-HD contained 60 μg hemagglutinin per strain of the US Vaccines and Related Biological Products Advisory Committee-recommended Northern Hemisphere 2017–2018 A/H1N1, A/H3N2, B Yamagata-lineage, and B Victoria-lineage strains in a 0.7-mL sterile suspension. IIV4-SD contained 15 μg hemagglutinin per strain of the National Institute of Infectious Diseases of Japan-recommended Northern Hemisphere 2017–2018 A/H1N1, A/H3N2, B Yamagata-lineage, and B Victoria-lineage strains in a 0.5-mL sterile suspension. Both vaccines were split-virion and preservative-free. Antigen content and strains in IIV4-HD and IIV4-SD are summarized in Table 1. Hemagglutinin content in the vaccines was measured by single radial immunodiffusion assay.

**Table 1.** Vaccines and strains.

Content	IIV4-HD	IIV4-SD
Administration	Intramuscular or subcutaneous injection	Subcutaneous injection
Hemagglutinin/strain	60 μg	15 μg
Formulation	US Vaccines and Related Biological Products Advisory Committee NH 2017–2018 ^a^	National Institute of Infectious Diseases of Japan NH 2017–2018 ^a^
A/H1N1 strain	A/Michigan/45/2015 (NYMC X-275)	A/Singapore/GP1908/2015 (IVR-180) pdm09 (“A/H1N1-like”)
A/H3N2 strain	A/Hong Kong/4801/2014 (NYMC X-263B)	A/Hong Kong/4801/2014 (NYMC X-263) (“A/H3N2-like”)
B Yamagata-lineage strain	B/Phuket/3073/2013	B/Phuket/3073/2013
B Victoria-lineage strain	B/Brisbane/60/2008	B/Texas/2/2013 (“B Victoria lineage-like”)

Abbreviations: IIV4-HD, high-dose quadrivalent inactivated influenza vaccine; IIV4-SD, standard-dose quadrivalent inactivated influenza vaccine; NH, Northern Hemisphere.

^a ^Strains chosen from the World Health Organization list of recommended strains for the 2017–2018 NH season

### Randomization

A computer-generated randomization list was generated and provided by the sponsor and used for labeling and packaging. In a first cohort, 10 participants were enrolled (cohort 1) and assigned in a 1:1 ratio by block randomization to receive IIV4-HD by IM or SC injection. After review of the local and systemic adverse events (AEs) occurring within 7 days post-vaccination by the sponsor’s safety review team, 165 participants were enrolled in a second cohort (cohort 2) and assigned in a 1:1:1 ratio by block randomization to receive IIV4-HD by IM injection, IIV4-HD by SC injection, or IIV4-SD by SC injection, with stratification by age (<75, ≥75 years), sex, and site. Site staff were provided with vaccine dose numbers via interactive response technology. Randomization codes were kept securely in the interactive response technology and an internal system.

### Blinding

An unblinded administrator at each site administered the vaccine. The vaccine administrator was not involved in any of the blinded study assessments. Investigators or delegates in charge of safety assessments, the trial staff who collected the safety data, and the laboratory personnel who analyzed the blood samples did not know which product was administered. Participants were blindfolded during vaccination to prevent them from identifying which vaccine was administered.

### Safety assessments

Unsolicited AEs, serious adverse events (SAEs), and solicited reactions were defined as described in the International Conference for Harmonization E2A Guideline for Clinical Safety Data Management: Definitions and Standards for Expedited Reporting.^21^ Solicited reactions were recorded by participants for 7 days after the vaccination using a diary card and unsolicited AEs were recorded by investigators up to the end of the study (day 28–35).

Injection-site erythema, swelling, induration and bruising were scored as grade 1 if 25–50 mm in diameter, grade 2 if 51–100 mm in diameter, and grade 3 if > 100 mm in diameter. Fever was scored as grade 1 if 100.4–101.1°F (38.0–38.4°C), grade 2 if 101.2–102.0°F (38.5–38.9°C), and grade 3 if ≥ 102.1°F (≥ 39.0°C); all other solicited reactions were scored as grade 1 if transient, requiring minimal therapeutic intervention, did not interfere with daily activities; grade 2, if they required additional therapeutic intervention, interfered with daily activities but posed no significant permanent risk; and grade 3 if they interrupted usual daily activities, significantly affected clinical status, or required intensive therapeutic intervention.

Unsolicited systemic AEs within the first 30 min after vaccination were recorded as immediate unsolicited systemic AEs. SAEs included any medical occurrence resulting in death, was life-threatening, required inpatient hospitalization or prolongation of existing hospitalization, resulted in persistent or significant disability/incapacity, was a congenital anomaly/birth defect, or was an important medical event according to the investigator’s judgment. AEs of special interest included new onset of Guillain-Barré syndrome, encephalitis/myelitis (including transverse myelitis), Bell’s palsy, optic neuritis, and brachial neuritis. For all AEs and SAEs, investigators recorded duration and the potential relationship to vaccination.

An early safety data review was performed by the sponsor after 7 days of post-vaccination safety data were available for cohort 1. The review included immediate reactions, solicited reactions, unsolicited AEs, SAEs, and AEs of special interest occurring within 7 days after vaccination. The study could have been put on hold or terminated if any of the following were detected during the review: ≥1 death or other SAE considered related to the vaccination; ≥40% of participants experiencing a grade 3 solicited reaction persisting ≥48 h; ≥20% of participants experiencing a grade 3 unsolicited, non-serious reaction persisting for ≥48 h and reported as related by the investigator.

### Hemagglutination inhibition antibody titers

All subjects provided a pre-vaccination (baseline) blood sample at day 0 and a post-vaccination blood sample on day 28–35 for HAI testing against US- or Japanese-recommended strains (Table 1), including A/Michigan/45/2015 (NYMC X-275)(H1N1), A/Hong Kong/4801/2014 (NYMC X-263B)(H3N2), B/Brisbane/60/2008, B/Phuket/3073/2013, A/Singapore/GP1908/2015(IVR-180) pdm09 (H1N1), A/Hong Kong/4801/2014 (NYMC X-263)(H3N2), and B/Texas/2/2013. As described previously,^22^ anti-influenza antibodies were measured using a hemagglutination inhibition (HAI) assay. Briefly, test and control sera were incubated with Type III neuraminidase to eliminate nonspecific inhibitors and then with a red blood cell suspension to adsorb spontaneous anti-species agglutinins. Ten two-fold dilutions of each sera were mixed and incubated with 4 hemagglutination units/25 µL of influenza virus. A red blood cell suspension was added to the mixture and, following incubation, titers were recorded as the highest serum dilution in which complete inhibition of hemagglutination occurred. If the lowest/first serum dilution used in the assay (1:10) did not result in complete inhibition of hemagglutination, the serum HAI titer was reported as < 10. If the highest/last serum dilution used in the assay (1:10,240) exhibited complete inhibition of hemagglutination, the serum HAI titer was reported as ≥ 10,240. The primary endpoints for the evaluation of immunogenicity were HAI antibody titers obtained on day 28 and seroconversion, defined as either (i) a HAI titer < 10 (1/dilution) at day 0 and a post-vaccination (day 28–35) HAI titer ≥ 40 or (ii) a HAI titer ≥ 10 at day 0 and a ≥ 4-fold increase in HAI titer between day 0 and post-vaccination.

### Statistical analysis

Because only descriptive analyses were performed for this study, no sample size calculation was made. Safety events were analyzed in the safety analysis set, defined as all participants who received a study vaccine. Immunogenicity was analyzed in the immunogenicity analysis set, defined as all participants in cohort 2 completing the study according to protocol. The 95% confidence intervals (CIs) for the geometric mean titers (GMTs) were calculated using a normal approximation of log-transformed titers. The 95% CIs of GMT ratios of each IIV4-HD group (IM or SC) compared to the IIV4-SD group (SC) were calculated using normal approximation of log-transformed titers. The 95% CIs for the proportions were calculated using the Clopper-Pearson method. Differences in the seroconversion rates between each IIV4-HD group (IM or SC) and the IIV4-SD group (SC) were computed along with the 2-sided 95% CIs by the Newcombe-Wilson method without continuity correction.

Statistical analyses were performed using SAS version 9.4 or later (SAS Institute, Cary, NC, USA). No search for outliers and no imputations were performed, although unsolicited AEs were considered as related if the investigator’s assessment of the relationship was missing.

## Results

### Participants

**Figure 1.**
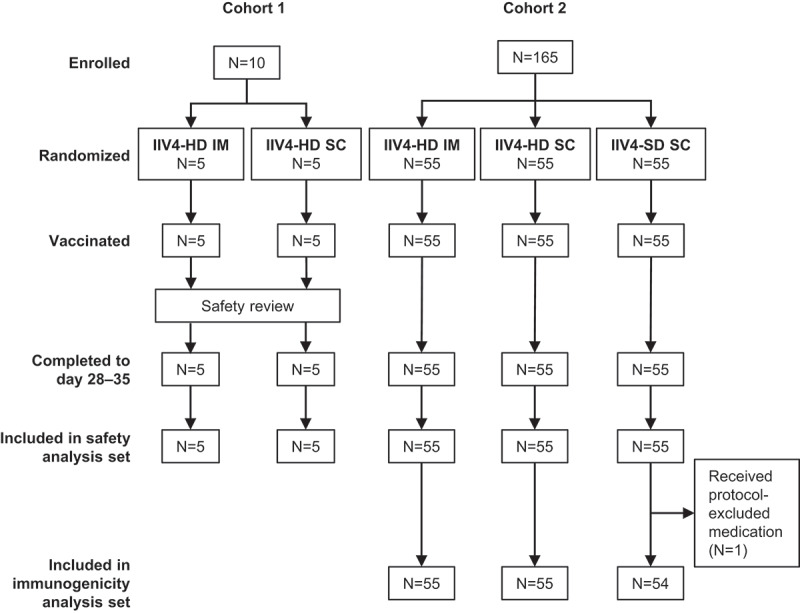
Study design and participant disposition. The first 10 participants enrolled (cohort 1) were assigned in a 1:1 ratio to receive IIV4-HD by IM or SC injection. After review of the local and systemic adverse events (AEs) occurring within 7 days post-vaccination by the sponsor’s safety review team, the remaining 165 participants enrolled (cohort 2) were assigned in a 1:1:1 ratio to receive IIV4-HD by IM injection, IIV4-HD by SC injection, or IIV4-SD by SC injection. Safety analysis was conducted for all participants who received a study vaccine (safety analysis set). Immunogenicity analysis was conducted for all participants in cohort 2 completing the study according to protocol (immunogenicity analysis set). Abbreviations: IIV4-HD, high-dose quadrivalent inactivated influenza vaccine; IIV4-SD, standard-dose quadrivalent inactivated influenza vaccine; IM, intramuscular; SC, subcutaneous.

As planned, the study recruited 175 adults ≥65 years of age (Figure 1) between September 15 and November 28, 2017 in two cohorts. A total of 60 participants were randomized to IIV4-HD by IM injection (IIV4-HD IM), 60 to IIV4-HD by SC injection (IIV4-HD SC), and 55 to IIV4-SD by SC injection (IIV4-SD SC). All participants were vaccinated as randomized and completed the study according to protocol, although one participant who received IIV4-SD by subcutaneous injection was excluded from the immunogenicity analysis set because of taking a prohibited medication before day 28–35 to treat a SAE.

All participants in this study were Asian, and age, sex, and body mass indices were similar in the three vaccine groups (Table 2). Proportions of participants vaccinated for influenza the previous year were similar in the IIV4-HD SC (31.7%) and IIV4-SD SC (30.9%) groups but lower in the IIV4-HD IM group (16.7%).

**Table 2.** Participant demographics.

	IIV4-HD IM	IIV4-HD SC	IIV4-SD SC
Characteristic	N = 60	N = 60	N = 55
**Age (years), mean (SD)**	70.2 (3.6)	70.6 (3.5)	69.9 (3.8)
**Age group, n (%)**			
<75 years	51 (85.0)	50 (83.3)	46 (83.6)
≥75 years	9 (15.0)	10 (16.7)	9 (16.4)
**Sex, n (%)**			
Male	32 (53.3)	33 (55.0)	30 (54.5)
Female	28 (46.7)	27 (45.0)	25 (45.5)
**Race, n (%)**			
Asian	60 (100.0)	60 (100.0)	55 (100.0)
**Body mass index (kg/m^2^), mean (SD)**	23.43 (2.89)	24.09 (2.85)	23.51 (2.61)
**Influenza vaccination the previous season, n (%)**	10 (16.7)	19 (31.7)	17 (30.9)

Values are for the safety analysis set. Abbreviations: IIV4-HD, high-dose quadrivalent inactivated influenza vaccine; IIV4-SD, standard-dose quadrivalent inactivated influenza vaccine; IM, intramuscular; SC, subcutaneous; SD, standard deviation.

### Solicited reactions

**Figure 2.**
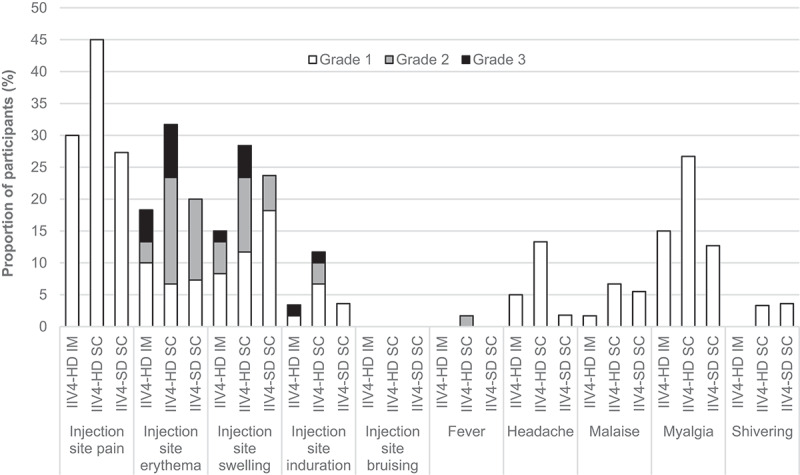
Solicited reactions. Participants recorded solicited reactions for 7 days after vaccination. Injection-site erythema, swelling, induration and bruising were grade 1 if 25–50 mm in diameter, grade 2 if 51–100 mm in diameter, and grade 3 if >100 mm in diameter. Fever was grade 1 if 100.4–101.1°F (38.0–38.4°C), grade 2 if 101.2–102.0°F (38.5–38.9°C), and grade 3 if ≥102.1°F (≥39.0°C). All other solicited reactions were grade 1 if transient, required minimal therapeutic intervention, and did not interfere with daily activities; grade 2 if they required additional therapeutic intervention or interfered with daily activities but posed no significant permanent risk, and grade 3 if they interrupted usual daily activities, significantly affected clinical status, or required intensive therapeutic intervention. Values are for the safety analysis set (N = 60 for IIV4-HD IM, N = 60 for IIV4-HD SC, and N = 55 for IIV4-SD SC). Abbreviations: IIV4-HD, high-dose quadrivalent inactivated influenza vaccine; IIV4-SD, standard-dose quadrivalent inactivated influenza vaccine; IM, intramuscular; SC, subcutaneous.

The most frequently reported solicited injection-site reaction was pain, followed by erythema and swelling (Figure 2). The most frequently reported solicited systemic reaction was myalgia, followed by headache and malaise. Most solicited reactions were of grade 1 or 2 intensity, started within the first 3 days after vaccination (Supplementary table S2), and resolved spontaneously within 7 days of onset (Supplementary table S3). Except for shivering, which was reported by few participants, and injection-site bruising, which was not reported, proportions reporting each solicited reaction were highest for the IIV4-HD SC group. Fever was reported in only one subject in the IIV4-HD SC group.

### Unsolicited adverse events

Treatment-related unsolicited AEs, mostly injection-site pruritus, were reported more often in participants vaccinated SC than IM, although in fewer than 10% of the participants in each group (Table 3). No immediate unsolicited AEs, AEs of special interest, or deaths were reported. A single SAE of sudden hearing loss 3 days after vaccination was reported for a participant in the IIV4-SD SC group. The event was not considered related to the vaccination.

**Table 3.** Unsolicited adverse events within 28 days after vaccination.

	Participants with the event, n (%)		
	IIV4-HD IM	IIV4-HD SC	IIV4-SD SC
Event	N = 60	N = 60	N = 55
Immediate unsolicited AE	0 (0.0)	0 (0.0)	0 (0.0)
Unsolicited AE	4 (6.7)	4 (6.7)	8 (14.5)
Treatment-related unsolicited AE	1 (1.7)^a^	3 (5.0)^b^	4 (7.3)^c^
AE leading to study discontinuation	0 (0.0)	0 (0.0)	0 (0.0)
SAE	0 (0.0)	0 (0.0)	1 (1.8)^d^
Death	0 (0.0)	0 (0.0)	0 (0.0)
AE of special interest^e^	0 (0.0)	0 (0.0)	0 (0.0)

Values are for the safety analysis set. Abbreviations: AE, adverse event IIV4-HD, high-dose quadrivalent inactivated influenza vaccine; IIV4-SD, standard-dose quadrivalent inactivated influenza vaccine; IM, intramuscular; SAE, serious adverse event; SC, subcutaneous.

^a^Injection-site pruritus (n = 1)

^b^Injection-site pruritus (n = 3)

^c^Injection-site pruritus (n = 1), oropharyngeal discomfort (n = 1), oropharyngeal pain (n = 1), soft feces (n = 1)

^d^Sudden hearing loss 3 days after vaccination, not considered related to the vaccination

^e^Included new onset of Guillain-Barré syndrome, encephalitis/myelitis (including transverse myelitis), Bell’s palsy, optic neuritis, and brachial neuritis

### HAI antibody response

Before vaccination (day 0), most subjects were seropositive (HAI titer ≥10), although HAI GMTs were similar between IIV4-HD and IIV4-SD for all strains (Supplementary table S4). Between baseline and day 28–35 post-vaccination, HAI titers increased for all strains in all vaccine groups by at least 2-fold and in most cases by at least 4-fold (Table 4). The highest geometric mean post-vaccination/pre-vaccination HAI titer ratios were for strains A/H1N1 (16.00 for IIV4-HD IM, 9.25 for IIV4-HD SC, and 6.56 for IIV4-SD SC) and A/H3N2 (16.93 for IIV4-HD IM, 8.31 for IIV4-HD SC, and 4.85 for IIV4-SD SC). HAI GMTs were similar between male and female participants (data not shown).

**Table 4.** Post-/pre-vaccination HAI GMT ratios.

Strain	Post-/pre-vaccination HAI GMT ratio (95% CI)		
	IIV4-HD IM	IIV4-HD SC	IIV4-SD SC
	N = 55	N = 55	N = 54
A/H1N1	16.00 (10.19, 25.11)	9.25 (6.11, 14.00)	6.56 (4.36, 9.86)
A/H1N1-like	9.25 (6.67, 12.82)	6.34 (4.79, 8.38)	5.13 (3.67, 7.17)
A/H3N2	16.93 (10.99, 26.10)	8.31 (5.54, 12.46)	4.85 (3.08, 7.63)
A/H3N2-like	12.36 (8.03, 19.01)	7.42 (4.98, 11.05)	4.56 (2.89, 7.19)
B Yamagata	7.51 (4.93, 11.45)	4.68 (3.34, 6.56)	3.11 (2.29, 4.24)
B Victoria	10.69 (7.05, 16.21)	6.92 (4.79, 9.99)	2.88 (2.08, 3.99)
B Victoria-like	8.26 (5.74, 11.89)	5.55 (3.97, 7.76)	2.67 (2.00, 3.57)

Values are for the immunogenicity analysis set. Abbreviations: CI, confidence interval; GMT, geometric mean titer; HAI, hemagglutination inhibition; IIV4-HD, high-dose quadrivalent inactivated influenza vaccine; IIV4-SD, standard-dose quadrivalent inactivated influenza vaccine; IM, intramuscular; SC, subcutaneous

#### Comparison of IIV4-HD and IIV4-SD

**Figure 3.**
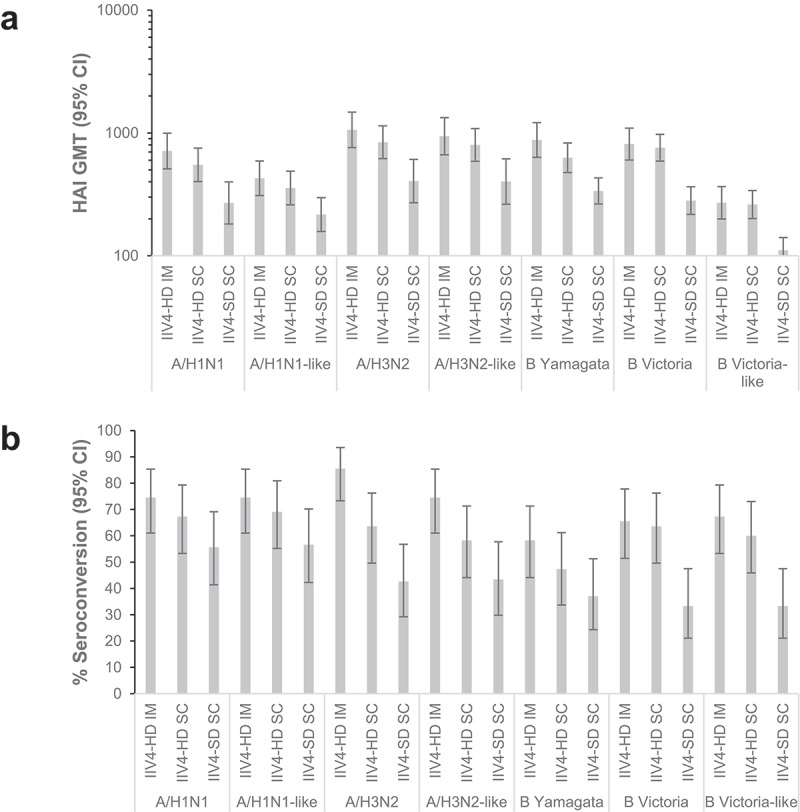
Post-vaccination HAI GMTs (a) and seroconversion rates (b). HAI titers were measured on day 0 and 28–35 days after vaccination for the seven influenza strains included in IIV4-HD and IIV4-SD. The three strains in IIV4-SD not identical to those in IIV4-HD are indicated as “-like” strains. (A) Post-vaccination HAI GMTs for each vaccination group 28–35 days after vaccination. (B) Proportions of patients in each group seroconverting. Seroconversion was defined as a HAI titer <10 (1/dilution) at day 0 and a post-injection titer ≥40 at day 28–35 or as a HAI titer ≥10 at day 0 and a ≥ 4-fold increase in HAI titer at day 28–35. Error bars indicate 95% CIs. The 95% CIs for the GMTs were calculated using a normal approximation of log-transformed titers, and the 95% CIs for the proportions were calculated using the Clopper-Pearson method. Values are for the immunogenicity analysis set (N = 55 for IIV4-HD IM, N = 55 for IIV4-HD SC, and N = 54 for IIV4-SD SC). Abbreviations: CI, confidence interval; GMT, geometric mean titer; HAI, hemagglutination inhibition; IIV4-HD, high-dose quadrivalent inactivated influenza vaccine; IIV4-SD, standard-dose quadrivalent inactivated influenza vaccine; IM, intramuscular; SC, subcutaneous.

Post-vaccination HAI GMTs were higher for IIV4-HD than for IIV4-SD, irrespective of administration route (IM or SC) or whether the US- or Japanese-recommended strains were tested (Figure 3a). Ratios of post-vaccination HAI GMTs between IIV4-HD and IIV4-SD ranged from 1.98 to 2.89 for the IIV4-HD IM group, and from 1.65 to 2.70 for the IIV4-HD SC group (Supplementary table S5). Similarly, for all strains, point estimates for seroconversion rates for both the IIV4-HD IM and IIV4-HD SC groups were higher than for the IIV4-SD SC group, irrespective of whether the US or Japanese strains were tested. (Figure 3b).

#### Comparison of IM and SC injection

Point estimates of post-vaccination HAI GMTs higher for the IIV4-HD IM group than for the IIV4-HD SC group (Figure 3a). Ratios of post-vaccination HAI GMTs for IIV4-HD IM vs. IIIV4-HD SC ranged from 1.03 to 1.40 (Supplementary table S6). Similarly, for all strains, point estimates for seroconversion rates were also higher for the IIV4-HD IM group than for the IIV4-HD SC group (Figure 3b).

#### Influence of strains tested

HAI GMTs for A/H1N1 and B Victoria-lineage strains (Figure 3a) tended to be higher when immunogenicity was measured using the US-recommended than using the Japanese-recommended strains, although seroconversion rates were similar (Figure 3b).

## Discussion

This study showed that IIV4-HD was well tolerated and highly immunogenic in healthy adults in Japan ≥65 years of age. No important safety events were detected for IIV4-HD. Although this study was not designed or powered to make statistical comparisons between IIV4-HD and IIV4-SD, the results are similar to those from studies comparing IIV3-HD and IIV3-SD in this population.^14,23,24^

Because influenza vaccines are typically administered by SC injection in Japan and IM injection in the US and many other countries, this study assessed the immunogenicity and safety of IIV4-HD administered by both routes. HAI antibody responses appeared higher and reactogenicity lower by IM administration than by SC administration. Thus, despite a higher antigen dose, the reactogenicity of IIV4-HD administered by IM injection was similar to that of IIV4-SD administered SC. Few other studies have directly compared SC and IM administration of influenza vaccines. A study in 120 adults 20 to 40 years of age in Japan found higher HAI antibody responses and fewer local adverse events following IM than SC administration of an adjuvanted influenza vaccine.^25^ Also, a study in 720 Australian adults ≥65 years found that a standard-dose split-virion trivalent inactivated influenza vaccine induced higher HAI antibody titers and fewer local reactions when administered by IM injection than SC injection.^26^ Although the immunogenicity differences in the Australian study were limited to women, sex did not appear to affect HAI antibody responses in the current study. The greater reactogenicity by SC injection is thought to be due to slower absorption of substances from the injection site, resulting in greater antigen processing by the antigen-presenting cells in the SC subcutaneous tissue and therefore greater inflammation.^27,28^ Despite some differences in reactogenicity and immunogenicity, IIV4-HD was well tolerated and highly immunogenic when administered by SC or IM injection.

IIV4-HD included the strains recommended by the US Vaccines and Related Biological Products Advisory Committee, whereas IIV4-SD included the strains recommended by the National Institute of Infectious Diseases of Japan. Although both the US and Japan used the WHO-recommended list of “-like” strains for influenza vaccines and the strains were from the same clades, three of four strains were not identical between the two vaccines. For all vaccine groups, HAI antibody titers were therefore measured for all seven strains. Irrespective of which strains were used for immunogenicity testing, conclusions were the same, with only small differences in HAI antibody titers.

In this study, 26.3% of the participants were vaccinated for influenza the previous year. By comparison, estimated influenza vaccination rates for Japanese adults ≥65 years of age were 50% in 2014 and 51% in 2015.^29^ The lower rate in the current study might be because of the limited sample and the inclusion of only healthy, community-dwelling individuals. Regardless, coverage in Japan appears to be below the World Health Organization target of 75% for this age group.^30^

An important limitation of this study is that it was not designed to make statistical comparisons between groups on immunogenicity or safety, and it was too small to detect low-frequency safety events. Rather, the study was primarily designed to confirm tolerability and immunogenicity and to provide data for designing future clinical trials. Additional studies in larger populations will be conducted to confirm these findings. Nonetheless, the current immunogenicity findings, coupled with the results of other studies comparing the efficacy and effectiveness of high- and standard-dose inactivated influenza vaccines,^13,17–19,31^ suggest that IIV4-HD can further improve protection against influenza infection in adults ≥65 years of age. Finally, this study included only healthy, community-dwelling older adults. Further study will be required to determine whether the findings apply to frail or institutionalized individuals.

In conclusion, this study showed that IIV4-HD can be safely administered by either IM or SC injection and that it is highly immunogenic against both the US and Japanese strains in adults ≥65 years of age. The results also add to the evidence that high-dose influenza vaccines are more immunogenic than standard-dose vaccines in this age group.^24^ Accordingly, IIV4-HD is predicted to provide better protection than IIV4-SD against influenza infection in adults ≥65 years of age.

## Data sharing statement

Qualified researchers may request access to patient level data and related study documents including the clinical study report, study protocol with any amendments, blank case report form, statistical analysis plan, and dataset specifications. Patient level data will be anonymized and study documents will be redacted to protect the privacy of our trial participants. Further details on Sanofi’s data sharing criteria, eligible studies, and process for requesting access can be found at: https://www.clinicalstudydatarequest.com/.
