# Supplementary material for: Immunogenicity and safety of high-dose quadrivalent influenza vaccine in Japanese adults ≥65 years of age: a randomized controlled clinical trial
Source: Hum Vaccin Immunother. 2019 Nov 19;16(4):858–66. doi: 10.1080/21645515.2019.1677437 (PMC7227668; doi:10.1080/21645515.2019.1677437)
Supplement: Supplemental Material [file khvi-16-04-1677437-s001.zip › QHD00008 ms_Table S1_for submission.docx]

**Supplementary** **table S1. Exclusion criteria**

| **Exclusion** | **Time frame** |
| --- | --- |
| Received any live vaccine | Past 27 days |
| Received any inactivated vaccine | Past 6 days |
| Planned to receive a vaccine | Prior to 28 days after the study vaccination |
| Received immune globulins, blood, or blood-derived products | Past 3 months |
| Known or suspected congenital or acquired immunodeficiency | Any |
| Received immunosuppressive therapy | Past 6 months |
| Received long-term systemic corticosteroid therapy (prednisone or equivalent) for more than 2 consecutive weeks | Past 3 months |
| Known systemic hypersensitivity to any of the vaccine components, or history of a life-threatening reaction to the vaccines used in the trial or to a vaccine containing any of the same substances | Any |
| Thrombocytopenia or bleeding disorder contraindicating intramuscular vaccination | Any |
