# Supplementary material for: Immunogenicity and safety of high-dose quadrivalent influenza vaccine in Japanese adults ≥65 years of age: a randomized controlled clinical trial
Source: Hum Vaccin Immunother. 2019 Nov 19;16(4):858–66. doi: 10.1080/21645515.2019.1677437 (PMC7227668; doi:10.1080/21645515.2019.1677437)
Supplement: Supplemental Material [file khvi-16-04-1677437-s001.zip › QHD00008 ms_Table S2_for submission.docx]

**Supplementary** **table S2. Onset of solicited reactions**

| **Solicited reaction** | **Time of onset** | **% of participants (95% CI)** | | |
| --- | --- | --- | --- | --- |
|  |  | **IIV4-HD IM** | **IIV4-HD SC** | **IIV4-SD SC** |
|  |  | **N=60** | **N=60** | **N=55** |
| Injection-site pain | Any | 30.0 (18.8, 43.2) | 45.0 (32.1, 58.4) | 27.3 (16.1, 41.0) |
|  | Day 0–3 | 30.0 (18.8, 43.2) | 45.0 (32.1, 58.4) | 25.5 (14.7, 39.0) |
|  | Day 4–7 | 0.0 (0.0, 6.0) | 0.0 (0.0, 6.0) | 1.8 (0.0, 9.7) |
|  |  |  |  |  |
| Injection-site erythema | Any | 18.3 (9.5, 30.4) | 31.7 (20.3, 45.0) | 20.0 (10.4, 33.0) |
|  | Day 0–3 | 18.3 (9.5, 30.4) | 31.7 (20.3, 45.0) | 20.0 (10.4, 33.0) |
|  | Day 4–7 | 0.0 (0.0, 6.0) | 0.0 (0.0, 6.0) | 0.0 (0.0, 6.5) |
|  |  |  |  |  |
| Injection-site swelling | Any | 15.0 (7.1, 26.6) | 28.3 (17.5, 41.4) | 23.6 (13.2, 37.0) |
|  | Day 0–3 | 15.0 (7.1, 26.6) | 28.3 (17.5, 41.4) | 23.6 (13.2, 37.0) |
|  | Day 4–7 | 0.0 (0.0, 6.0) | 0.0 (0.0, 6.0) | 0.0 (0.0, 6.5) |
|  |  |  |  |  |
| Injection-site induration | Any | 3.3 (0.4, 11.5) | 11.7 (4.8, 22.6) | 3.6 (0.4, 12.5) |
|  | Day 0–3 | 3.3 (0.4, 11.5) | 11.7 (4.8, 22.6) | 3.6 (0.4, 12.5) |
|  | Day 4–7 | 0.0 (0.0, 6.0) | 0.0 (0.0, 6.0) | 0.0 (0.0, 6.5) |
|  |  |  |  |  |
| Injection-site bruising | Any | 0.0 (0.0, 6.0) | 0.0 (0.0, 6.0) | 0.0 (0.0, 6.5) |
|  | Day 0–3 | 0.0 (0.0, 6.0) | 0.0 (0.0, 6.0) | 0.0 (0.0, 6.5) |
|  | Day 4–7 | 0.0 (0.0, 6.0) | 0.0 (0.0, 6.0) | 0.0 (0.0, 6.5) |
|  |  |  |  |  |
| Fever | Any | 0.0 (0.0, 6.0) | 1.7 (0.0, 8.9) | 0.0 (0.0, 6.5) |
|  | Day 0–3 | 0.0 (0.0, 6.0) | 0.0 (0.0, 6.0) | 0.0 (0.0, 6.5) |
|  | Day 4–7 | 0.0 (0.0, 6.0) | 1.7 (0.0, 9.1) ^a^ | 0.0 (0.0, 6.5) |
|  |  |  |  |  |
| Headache | Any | 5.0 (1.0, 13.9) | 13.3 (5.9, 24.6) | 1.8 (0.0, 9.7) |
|  | Day 0–3 | 5.0 (1.0, 13.9) | 8.3 (2.8, 18.4) | 0.0 (0.0, 6.5) |
|  | Day 4–7 | 0.0 (0.0, 6.0) | 5.0 (1.0, 13.9) | 1.8 (0.0, 9.7) |
|  |  |  |  |  |
| Malaise | Any | 1.7 (0.0, 8.9) | 6.7 (1.8, 16.2) | 5.5 (1.1, 15.1) |
|  | Day 0–3 | 1.7 (0.0, 8.9) | 5.0 (1.0, 13.9) | 3.6 (0.4, 12.5) |
|  | Day 4–7 | 0.0 (0.0, 6.0) | 1.7 (0.0, 8.9) | 1.8 (0.0, 9.7) |
|  |  |  |  |  |
| Myalgia | Any | 15.0 (7.1, 26.6) | 26.7 (16.1, 39.7) | 12.7 (5.3, 24.5) |
|  | Day 0–3 | 15.0 (7.1, 26.6) | 26.7 (16.1, 39.7) | 12.7 (5.3, 24.5) |
|  | Day 4–7 | 0.0 (0.0, 6.0) | 0.0 (0.0, 6.0) | 0.0 (0.0, 6.5) |
|  |  |  |  |  |
| Shivering | Any | 0.0 (0.0, 6.0) | 3.3 (0.4, 11.5) | 3.6 (0.4, 12.5) |
|  | Day 0–3 | 0.0 (0.0, 6.0) | 1.7 (0.0, 8.9) | 3.6 (0.4, 12.5) |
|  | Day 4–7 | 0.0 (0.0, 6.0) | 1.7 (0.0, 8.9) | 0.0 (0.0, 6.5) |

Values are for the safety analysis set. Abbreviations: CI, confidence interval; IIV4-HD, high-dose quadrivalent inactivated influenza vaccine; IIV4-SD, standard-dose quadrivalent inactivated influenza vaccine; IM, intramuscular; SC, subcutaneous.

^a^ Data unavailable for one participant
