# Supplementary material for: Immunogenicity and safety of high-dose quadrivalent influenza vaccine in Japanese adults ≥65 years of age: a randomized controlled clinical trial
Source: Hum Vaccin Immunother. 2019 Nov 19;16(4):858–66. doi: 10.1080/21645515.2019.1677437 (PMC7227668; doi:10.1080/21645515.2019.1677437)
Supplement: Supplemental Material [file khvi-16-04-1677437-s001.zip › QHD00008 ms_Table S4_revised_for submission.docx]

**Supplementary** **table S4. Pre-vaccination HAI GMTs and seropositivity rates**

|  | **IIV4-HD IM** | | **IIV4-HD SC** | | **IIV4-SD SC** | |
| --- | --- | --- | --- | --- | --- | --- |
|  | **N=55** | | **N=55** | | **N=54** | |
| **Strain** | **HAI GMT (95% CI)** | **Seropositive (%) ^a^** | **HAI GMT (95% CI)** | **Seropositive (%) ^a^** | **HAI GMT (95% CI)** | **Seropositive (%) ^a^** |
| A/H1N1 | 44.5 (29.4, 67.5) | 74.5 | 59.5 (39.7, 89.2) | 87.3 | 41.0 (27.7, 60.8) | 83.3 |
| A/H1N1-like | 46.2 (35.4, 60.4) | 96.4 | 56.2 (43.0, 73.5) | 100 | 42.1 (32.8, 54.1) ^b^ | 100.0 ^b^ |
| A/H3N2 | 62.6 (39.9, 98.1) | 85.5 | 101 (66.9, 152.6) | 90.9 | 83.7 (53.9, 129.9) | 90.7 |
| A/H3N2-like | 76.1 (49.6, 116.7) | 89.1 | 107.6 (71.4, 162.1) | 92.7 | 91.0 (57.7, 143.3) | 90.7 |
| B Yamagata | 116.8 (83.2, 163.9) | 98.2 | 134.1 (92.2, 195.0) | 96.4 | 108.2 (79.5, 147.1) | 98.1 |
| B Victoria | 76.1 (49.9, 115.9) | 89.1 | 109.6 (73.4, 163.8) | 94.5 | 97.6 (69.6, 137.0) | 100 |
| B Victoria-like | 32.7 (22.9, 46.7) | 80.0 | 47.1 (32.8, 67.6) | 90.9 | 41.6 (31.4, 55.1) | 90.7 |

Values are for the immunogenicity set. Abbreviations: CI, confidence interval; GMT, geometric mean titer; HAI, hemagglutination inhibition; IIV4-HD, high-dose quadrivalent inactivated influenza vaccine; IIV4-SD, standard-dose quadrivalent inactivated influenza vaccine; IM, intramuscular; SC, subcutaneous.

^a^ HAI titer ≥10

^b^ Data unavailable for one participant
