# Supplementary material for: Immunogenicity and safety of high-dose quadrivalent influenza vaccine in Japanese adults ≥65 years of age: a randomized controlled clinical trial
Source: Hum Vaccin Immunother. 2019 Nov 19;16(4):858–66. doi: 10.1080/21645515.2019.1677437 (PMC7227668; doi:10.1080/21645515.2019.1677437)
Supplement: Supplemental Material [file khvi-16-04-1677437-s001.zip › QHD00008 ms_Table S5_for submission.docx]

**Supplementary** **table S5. Comparison of post-vaccination HAI GMTs for IIV4-HD and IIV4-SD**

|  |  |  | **IIV4-HD/IIV4-SD** |
| --- | --- | --- | --- |
| **Vaccine and administration route** | **Strain** | **Post-vaccination HAI GMT (95% CI)** | **Ratio of post-vaccination HAI GMTs (95% CI)** |
| IIV4-HD IM (N=55) | A/H1N1 | 712.4 (509.7, 995.7) | 2.65 (1.59, 4.41) |
|  | A/H1N1-like | 427.6 (309.1, 591.5) | 1.98 (1.26, 3.10) |
|  | A/H3N2 | 1059.5 (759.5, 1478.0) | 2.61 (1.56, 4.38) |
|  | A/H3N2-like | 940.0 (664.3, 1330.1) | 2.34 (1.36, 4.01) |
|  | B Yamagata | 877.0 (632.9, 1215.3) | 2.60 (1.74, 3.90) |
|  | B Victoria | 813.2 (603.0, 1096.5) | 2.89 (1.95, 4.28) |
|  | B Victoria-like | 269.9 (199.4, 365.4) | 2.43 (1.66, 3.56) |
|  |  |  |  |
| IIV4-HD SC (N=55) | A/H1N1 | 550.2 (402.2, 752.5) | 2.04 (1.24, 3.36) |
|  | A/H1N1-like | 356.2 (260.0, 488.0) | 1.65 (1.06, 2.56) |
|  | A/H3N2 | 839.2 (617.7, 1140.0) | 2.07 (1.25, 3.41) |
|  | A/H3N2-like | 797.9 (586.8, 1084.9) | 1.98 (1.18, 3.32) |
|  | B Yamagata | 628.0 (475.4, 829.6) | 1.86 (1.29, 2.69) |
|  | B Victoria | 758.7 (589.5, 976.5) | 2.70 (1.88, 3.86) |
|  | B Victoria-like | 261.6 (201.1, 340.2) | 2.36 (1.66, 3.35) |
|  |  |  |  |
| IIV4-SD SC (N=54) | A/H1N1 | 269.1 (181.5, 399.0) |  |
|  | A/H1N1-like | 216.3 (157.4, 297.3) |  |
|  | A/H3N2 | 405.8 (270.5, 608.6) |  |
|  | A/H3N2-like | 402.3 (263.0, 615.4) ^a^ |  |
|  | B Yamagata | 336.9 (263.7, 430.3) |  |
|  | B Victoria | 281.5 (217.0, 365.0) |  |
|  | B Victoria-like | 111.0 (87.6, 140.7) |  |

Values are for the immunogenicity analysis set.: CI, confidence interval; GMT, geometric mean titer; HAI, hemagglutination inhibition; IIV4-HD, high-dose quadrivalent inactivated influenza vaccine; IIV4-SD, standard-dose quadrivalent inactivated influenza vaccine; IM, intramuscular; SC, subcutaneous.

^a^ Data unavailable for one participant
