# Supplementary material for: Immunogenicity and safety of high-dose quadrivalent influenza vaccine in Japanese adults ≥65 years of age: a randomized controlled clinical trial
Source: Hum Vaccin Immunother. 2019 Nov 19;16(4):858–66. doi: 10.1080/21645515.2019.1677437 (PMC7227668; doi:10.1080/21645515.2019.1677437)
Supplement: Supplemental Material [file khvi-16-04-1677437-s001.zip › QHD00008 ms_Table S6_for submission.docx]

**Supplementary** **table S6. Comparison of post-vaccination HAI GMTs for IIV4-HD administered by IM and SC injection**

|  |  |  | **IM route/SC route** |
| --- | --- | --- | --- |
| **Vaccine and administration route** | **Strain** | **Post-vaccination HAI GMT (95% CI)** | **Ratio of post-vaccination HAI GMTs (95% CI)** |
| IIV4-HD IM (N=55) | A/H1N1 | 712.4 (509.7, 995.7) | 1.29 (0.82, 2.04) |
|  | A/H1N1-like | 427.6 (309.1, 591.5) | 1.20 (0.77, 1.88) |
|  | A/H3N2 | 1059.5 (759.5, 1478.0) | 1.26 (0.81, 1.97) |
|  | A/H3N2-like | 940.0 (664.3, 1330.1) | 1.18 (0.74, 1.86) |
|  | B Yamagata | 877.0 (632.9, 1215.3) | 1.40 (0.91, 2.13) |
|  | B Victoria | 813.2 (603.0, 1096.5) | 1.07 (0.73, 1.58) |
|  | B Victoria-like | 269.9 (199.4, 365.4) | 1.03 (0.69, 1.53) |
|  |  |  |  |
| IIV4-HD SC (N=55) | A/H1N1 | 550.2 (402.2, 752.5) | - |
|  | A/H1N1-like | 356.2 (260.0, 488.0) | - |
|  | A/H3N2 | 839.2 (617.7, 1140.0) | - |
|  | A/H3N2-like | 797.9 (586.8, 1084.9) | - |
|  | B Yamagata | 628.0 (475.4, 829.6) | - |
|  | B Victoria | 758.7 (589.5, 976.5) | - |
|  | B Victoria-like | 261.6 (201.1, 340.2) | - |

Values are for the immunogenicity analysis set. Abbreviations: CI, confidence interval; GMT, geometric mean titer; HAI, hemagglutination inhibition; IIV4-HD, high-dose quadrivalent inactivated influenza vaccine; IM, intramuscular; SC, subcutaneous
